# Addressing post‐tuberculosis sequelae among people living with HIV: an unmet need

**DOI:** 10.1002/jia2.26439

**Published:** 2025-03-24

**Authors:** Vidya Mave, Samyra R. Cox, Akshay N. Gupte, Jonathan E. Golub

**Affiliations:** ^1^ Center for Infectious Diseases in India Johns Hopkins India Pune India; ^2^ School of Medicine Johns Hopkins University Baltimore Maryland USA; ^3^ Bloomberg School of Public Health Johns Hopkins University Baltimore Maryland USA; ^4^ School of Public Health Boston University Boston Massachusetts USA

1

In 2023, an estimated 10.8 million people fell ill with tuberculosis (TB) and 662,000 (6%) of them were living with HIV. Over the same period, an estimated 1.1 million people died with TB, and HIV co‐infection contributed to an additional 161,000 TB‐related deaths [1]. Since 2010, global TB incidence and related mortality have been gradually declining due to increased access to new diagnostics and expanding treatment options. However, there is growing evidence that TB survivors experience excess morbidity and mortality even after successfully completing treatment. We must begin focusing more on caring for the 155 million TB survivors worldwide who are struggling with the aftermath of TB disease [2]. The post‐TB sequelae that these survivors experience include TB recurrence, lung disease, cardiovascular disease, mental health issues and an overall decline in quality of life [3].

Screening TB survivors for recurrent TB could lead to major reductions in TB transmission and improvements in patient outcomes, particularly in high HIV‐prevalence countries which tend to have higher rates of TB recurrence [4]. Recent evidence suggests that periodic screening for TB survivors is a high‐yield approach for active case finding. The Targeted Universal Testing for TB study in South Africa found a new TB diagnosis among 12% of people with recent prior TB—higher than what was found among people living with HIV and among close contacts of people with TB in the study [5]. However, so far, the World Health Organization has only made a conditional recommendation for post‐TB screening due to limited evidence [6]. To help address this gap, results are forthcoming from the first active case‐finding trial targeting TB survivors and their household contacts, TB Aftermath [7]. More work is needed to optimize post‐TB screening so that it can be effective in a range of settings, including among people living with HIV. A priority is to identify post‐TB screening algorithms that are low‐cost but accurate and deployable in both clinic and community contexts, as well as efficient strategies that target the highest‐risk groups for TB recurrence, without deepening TB and HIV‐related stigma.

Comorbidities and non‐TB conditions affecting people with TB during TB treatment almost always persist beyond TB, and some of these conditions first develop as TB treatment concludes. Screening for and treating non‐TB conditions is, thus, necessary as early as possible to reduce post‐TB morbidity and mortality. Identifying optimal care strategies for multi‐morbidities during and after completion of TB treatment, including maintaining continued care and treatment of HIV, is also critical to optimize health outcomes. However, more research on the longer‐term health outcomes of TB beyond 24 months of follow‐up would help identify interventions and resources that national programmes need to manage the disease burden.

Despite treatment, as many as two‐thirds of TB survivors have impaired lung function that is associated with excess disability and mortality [8]. Post‐TB lung disease is highly heterogenous and can include airflow obstruction, restriction or a combination of both patterns [9]. Interestingly, people living with HIV have a lower burden and severity of post‐TB lung disease compared to people without HIV [10]. While the precise reasons for this are unclear, a dampened host inflammatory response in people living with HIV that limits the extent of TB‐associated lung injury has been hypothesized. Systematic comparisons of the immunological endotypes associated with persistent post‐TB lung injury among people living with and without HIV are needed to identify prognostic biomarkers and potential targets for immunomodulatory therapies to improve lung health and longevity. Importantly, children and adolescents treated for TB can also develop post‐TB respiratory disease, but research among this group remains under‐prioritized, particularly among children with HIV.

Among adult TB survivors, cardiovascular disease is estimated to account for nearly half of their excess mortality [11]. Yet, the phenotypic presentations and the underlying pathophysiology of TB‐associated cardiovascular disease remain largely unknown. Further, HIV is independently associated with cardiovascular disease [12]; and whether HIV modifies the association between TB and cardiovascular disease is unclear. A comprehensive evaluation of the natural history of TB‐associated cardiovascular disease and its pathophysiology would identify biomarkers for risk stratification and potential targets for immunomodulatory therapies.

Quality of life for people who have experienced TB is not only impaired by physical disability, but also catastrophic costs associated with TB, social stigma and mental health issues. TB‐related stigma is pervasive across high‐TB‐burden settings, and there is emerging evidence that perceived and experienced stigma affects TB survivors and their households for years beyond treatment. Mental health issues, including depression and anxiety, are common among people with TB. While these conditions often improve during TB treatment, untreated depression can lead to decreased quality of life and prolonged disability post‐treatment [3]. To improve TB survivors’ overall wellbeing, we need to refine integrated screening and care delivery models that encompass both medical and psychosocial care and that take a truly person‐centred approach. For example, peer‐led support models have been successful in the context of HIV, and should be further investigated and leveraged to help TB survivors navigate the complexities of post‐TB care.

HIV co‐infection amplifies the impact of the many challenges that people with TB face during and after their treatment. Prevalent comorbidities such as diabetes, hypertension, tobacco smoking and alcohol use among TB survivors are also common to people with HIV. Those experiencing both conditions are at elevated risk for TB recurrence compared to their counterparts without HIV and face similar and also unique challenges in the post‐treatment period. However, where HIV and TB care programmes have integrated care, this care is focused largely on the period of TB treatment. We now have the opportunity and the imperative to build on the HIV/TB integration model and begin to systematically screen TB survivors with HIV for post‐TB sequelae and strengthen their linkage to care.

In summary, increasing recognition of post‐TB sequelae is welcome, but further research should focus on early identification and optimal implementation strategies. Emerging research in this field will offer further insights, but more needs to be done, particularly among people with HIV who are disproportionately affected by TB and many of its long‐term consequences.

## COMPETING INTERESTS

The authors declare no competing interests.

## AUTHORS’ CONTRIBUTIONS

1

All authors equally contributed to this viewpoint.
